# The HONEST Cohort Study: Rationale and Design of a Nationwide Subcutaneous Implantable Cardioverter-Defibrillator Cohort

**DOI:** 10.1016/j.jacadv.2026.102604

**Published:** 2026-03-25

**Authors:** Rodrigue Garcia, Fawzi Kerkouri, Christelle Marquié, Warda Aoudjeghout, Fréderic Anselme, Pascal Defaye, Jérôme Hourdain, Christophe Leclercq, Pierre Mondoly, David Perrot, Nicolas Sadoul, Vincent Probst, Serge Boveda, Eloi Marijon, HONEST Investigators, Alexandre Doucy, Hassan Barake, Aziz Kneizeh, Didier Scarlatti, Fabien Squara, Sok-Sithikun Bun, Philippe Durand, Lara Dabiri, Philippe Ricard, Laurent Liprandi, Garret Gauthier, Folco Frattini, Yann Dagher Hayeck, Alaa Al Amoura, Bruno Maillier, Denis Gaty, Lilian Marty, Mohammed Benkaci Ali, Jean Claude Deharo, Jérome Hourdain, Baptiste Maille, Mickael Peyrol, Jérémie Barraud, Florian Baptiste, Jerome Taieb, Jerome Bouet, Gilles Macaluso, Alexis Mechulan, Sébastien Prevot, Ahmed Bouharaoua, Edouard Gitenay, Clement Bars, Julien Seitz, Laure Champ Rigot, Pierre Ollitrault, Paul-Ursmar Milliez, Arnaud Pellissier, Alain Lebon, Sophie Gomes, Pamela Damiano, Antoine Milhem, Cecile Duplantier, Cyril Goujeau, Isabelle Heurtebise, Vincent Ahfat, Lila Khris, Charles Guenancia, Géraldine Bertaux, Gabriel Laurent, Audrey Sagnard, Marie Fichot, Sylvain Bodi, Anne Quentin, Jonathan Lacaze Gadonneix, Jean Litalien, Mathieu Courtheix, Philippe Jarnier, Marc Badoz, Baptiste Favoulet, Guillaume Serzian, Hugues Zimmermann, Aurélien Miralles, Marie Pierre Chatel, Eric Ramiaramanana, Hervé Gorka, Maria Moldovan, Christophe Laure, Jacques Mansourati, Fawzi Kerkouri, Vincent Mansourati, Pierre Winum, Julien Roux, Pénélope Pujadas, Pierre Mondoly, Philippe Maury, Guillaume Domain, Serge Boveda, Nicolas Combes, Stéphane Combes, Christèle Cardin, Jean-Paul Albenque, Romain Cassagneau, Pierre Bordachar, Sylvain Ploux, Frédéric Sacher, Nicolas Derval, Olivier Cesari, Rim El Bouazzaoui, Adlane Zemmoura, Thien-Tri Cung, Frederic Cransac, Nicolas Clementy, Pierre Gallay, Maxime Pons, Jean Luc Pasquié, Mathieu Garnier, Nathalie Behar, Christophe Leclercq, Vincent Galand, Raphael Martins, Dominique Pavin, Frédéric Victor, Bertrand Pierre, Laurent Fauchier, Arnaud Bisson, Cyril Zakine, Christophe Loose, Akli Otmani, Pascal Defaye, Peggy Jacon, Adrien Carabelli, Sandrine Venier, Luc Petit, Xavier Dreyfus, Corina Moldovan, Antoine Da Costa, Cécile Romeyer-Bouchard, Jean-Baptiste Guichard, Jerome Thevenin, Vincent Probst, Jean Baptiste Gourraud, Antoine Andorin, Damien Minois, Daniel Gras, Cédric Giraudeau, Radu Moisei, René Gabriel Huguet, Julien Rischard, Soraya Anys, Benjamin Monteil, Sophie Le Page, Mouna Ben Kilani, Jean-Marc Dupuis, Frederic Treguer, Michel Merheb, Jean Pierre Chabert, Francois Lesaffre, Madeline Espinosa, Nicolas Luconi, Thibault Villemin, Raphael Sandras, Karim Bel Hadj, Jerome Schwartz, Arnaud Olivier, Daniel Beurrier, Pierre-Yves Zinzius, Nicolas Sadoul, Hugues Blangy, Luc Freysz, Christian De Chillou, Stéphane Evain, Eric Rendu, Pierre Khattar, Aude Zanutto, Marc Mielczarek, Julien Bertrand, Mathieu Becker, Pierre Houriez, Yves Guyomard, Aymeric Menet, Olivier Brimont, Karine Bauley, Stephane Dennetiere, Christelle Marquié, Charlotte Potelle, Didier Klug, Romain Sellier, Laura Forelle, Frédérique Mizon-Gérard, Arthur Vaksmann, Frederic Elmkies, Thierry Zerah, Eric Verbrugge, Aurélie Guiot, Marc Poueymidanette, Emilie Marcant, Claire Vanesson, Thibault Hus, Romain Eschalier, Frederic Jean, Grégoire Massoullié, Yannick Saludas, François Philippot, Antoine Roux, Maxime De Guillebon, Hugues Bader, Prune Gaillard, Philippe Couderc, Aurelien Hebrard, Nicolas Klotz, Julien Laborderie, Michel Lerecouvreux, Christian Demasles, Sorin Pripon, Michel Voglimacci, Dominique Celse, Philippe Lagrange, Ziad Khoueiry, Pierre Sultan, Georges Nadji, Mathieu Steinbach, Sebastien Bufflerin, Michel Chauvin, Alexandre Schatz, Laurence Jesel-Morel, Sophie Pynn, Sandrine Bellmont, Jacques Levy, Ronan Le Bouar, Serban Schiau, Lucien Diene, Francis Bessiere, Arnaud Dulac, Philippe Chevalier, Kevin Gardey, Cyril Durand, Alexis Durand Dubief, Hugo Brahic, Hervé Poty, Benjamin Gal, Julien Pineau, Samuel Chauveau, Olivier Garrier, Michael Attali, Samir Fareh, Mathieu Montoy, Pierre Lantelme, Paul Charles, Cédric Nguyen, Mathieu Amelot, Philippe Poret, Jean Christophe Amirault, Raoul Bacquelin, Pierre Frey, Didier Irles, Antoine Dompnier, Chrystelle Akret, Edouard Siméon, Olivier Villejoubert, Nicolas Mignot, Pierre Jorrot, Yamina Mouhoub, Lionel Ovart, Géraldine Vedrenne, Jacky Ollitrault, Denis Amet, David Perrot, Emilie Varlet, Pierre Baudinaud, Thomas Lavergne, Xavier Jouven, Séverine Philibert, Pauline Pinon, Tej Chalbia, Victor Waldmann, Eloi Marijon, Nicolas Badenco, Estelle Gandjbakhch, Guillaume Duthoit, Mikael Laredo, Xavier Waintraub, Anne Messali, Antoine Leenhardt, Vincent Algalarrondo, Fabrice Extramiana, Victor Waldmann, Damien Bonnet, Benedicte Godin, Frederic Anselme, Arnaud Savouré, Corentin Chaumont, Nathanael Auquier, Popescu Elena, Pierre Le Franc, Fanny Bouchinet, Cyrus Moini, Audrey Lefoulon, Mohamed Belhameche, Sana Sioua, Abdeslam Bouzeman, Cathy Bertrand, Franck Halimi, Thomas Chastre, Khadidja Belkir, Raphael Gdalia, Denis Amet, Jean Sylvain Hermida, Alexis Hermida, Akli Otmani, Maciej Kubala, Sarah Traulle, Denis Raguin, Marie Blaye-Felice, Philippe Rumeau, Pascal Chavernac, Marion Pouche, Nouredine El Hajjaji, Emilie Bastard, Isabelle Lecardonnel, Essia Lakhal-Ben Larbi, Gilles Cellarier, Raphaël Demoulin, Olivier Barthez, Jean Paul Faugier, Saida Cheggour, François Xavier Hager, Frédéric Ortuno, Olivier Billon, Rodrigue Garcia, Bruno Degand, François Le Gal, Benoit Guy Moyat, François Jourda, Stéphane Mourot, Renaud Fouché, Jerome Horvilleur, Laurent Fiorina, Jerome Lacotte, Fiorella Salerno, Salem Younsi, Mina Ait Said, Vladimir Manenti, Mohanad Mahfoud, Monteau Jacques, Vladimir Manenti, Dominique Bleinc, Christine Alonso, Arnaud Lazarus, Ghassan Moubarak, Olivier Thomas, Ardalan Sharifzadehgan, Alexandre Zhao, Christophe Juin, Vincent Kanczuga, Henri Broustet, Nicolas Combes, Alice Maltret, Abdelhamid Benounane, Xavier Copie, Olivier Piot, Walid Amara, Vanessa Abdou, Fabien Monsel, Nicolas Lellouche, Nathalie Elbaz, Segolene Rouffiac-Noel, Guillaume Galidie, Laurentiu Dorian Nitu, Gabriel Latcu, Bogdan Enache, Nicolas Hugues, Isabelle Lagrenade, Fabrice Demoniere, Inamo Jocelyn, Andréas Müssigbrodt, Olivier Geoffroy, Gael Clerici, François Wiart, Bruno Ulmer, Guillaume Kabalu, Olivier Axler

**Affiliations:** a University Hospital of Poitiers, Poitiers, France; b Centre investigation Clinique 1402, University Hospital of Poitiers, Poitiers, France; c Université Paris Cité, INSERM, U970, Paris Cardiovascular Research Centre, Paris, France; d University Hospital of Brest, Brest, France; e University Brest, Laboratoire ORPHY EA 4324, Brest, France; f University Hospital of Lille, Lille, France; g University Hospital of Rouen, Rouen, France; h University Hospital of Grenoble, Grenoble, France; i University Hospital of Marseille - La Timone, Marseille, France; j University Hospital of Rennes, Rennes, France; k University Hospital of Toulouse, Toulouse, France; l Division of Cardiology, European Georges Pompidou Hospital, Paris, France; m University Hospital of Nancy, Nancy, France; n University Hospital of Nantes, Nantes, France; o Pasteur Clinic, Toulouse, France; p Brussels University, VUB, Brussels, Belgium

**Keywords:** appropriate therapy, complications, hospitalization, mortality, remote monitoring, sudden death

## Abstract

The subcutaneous implantable cardioverter-defibrillator (S-ICD) was developed to reduce complications associated with transvenous leads while preserving efficacy in terminating malignant ventricular arrhythmias. However, current evidence supporting the S-ICD is primarily derived from industry-sponsored studies and highly specialized centers. This study aims to offer a comprehensive, nationwide, real-world assessment of all patients who underwent S-ICD implantation in France. The HONEST (coHOrte fraNcaise des dEfibrillateurs Sous cuTanés) cohort is a French nationwide, single-arm, observational ongoing study that enrolled all patients who underwent implantation of an S-ICD (EMBLEM, Boston Scientific) between October 2012 (the first implantation in France) and December 2019. Clinical endpoints are centrally adjudicated. Overall, 4,924 (77% males; 49.9 years) patients, representing 98.2% of all S-ICD implantations during the study period in France, were included. The HONEST study represents the first nationwide cohort evaluating S-ICD recipients to provide contemporary, real-world data. Its goal is to identify areas for improvement and refine strategies to prevent sudden cardiac death. (S-ICD French Cohort Study [HONEST]; NCT05302115)

## Highlights

• S-ICD reduces transvenous-lead complications, preserves arrhythmia termination efficacy, but evidence derives mainly from industry, specialized centers.
• The HONEST cohort enrolled 4,924 S-ICD patients, representing 98.2% of French implants (2012-2019).
• All patients were remotely monitored; outcomes—shocks, device complications, and mortality—were centrally adjudicated.
• HONEST—the first nationwide S-ICD cohort—leverages real-world data aiming to refine sudden cardiac death prevention strategies.

## Background and rationale

The need to improve current strategies for the prevention of sudden cardiac death (SCD), potentially through emerging technologies, is well recognized.^1^, ^2^, ^3^, ^4^, ^5^ While transvenous implantable cardioverter-defibrillators (ICDs) have proven effective in preventing SCD in high-risk patients,^6^^,^^7^ complications related to transvenous leads, such as infection and lead dysfunction, have prompted the development of the entirely subcutaneous ICD (S-ICD).^8^

**Figure 1:**
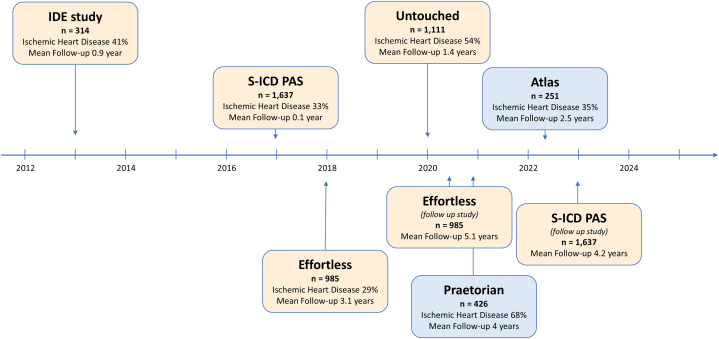
**Major Studies Assessing S-ICD Since 2013** Observational studies have a yellow background, and randomized control trials have a blue background. IDE = Investigational Device Exemption; PAS = Post Approval Study; S-ICD = subcutaneous implantable cardioverter-defibrillator.

Multiple studies have demonstrated the efficacy of the S-ICD in terminating life-threatening ventricular arrhythmias, while highlighting its lower risk of systemic infection and lead-related complications, compared with traditional transvenous ICDs.^9^^,^^10^ However, the S-ICD also presents limitations, most notably, the inability to provide bradycardia pacing or antitachycardia pacing, which may be a significant drawback for certain patients. Additionally, as with any novel device, concerns about hardware malfunctions and product recalls persist, potentially resulting in serious clinical consequences. These limitations underscore the importance of independent, rigorous postmarket surveillance to ensure long-term safety and efficacy.^11^^,^^12^ To date, much of the evidence supporting the S-ICD has been derived from industry-sponsored studies conducted in highly specialized centers, often involving modest sample sizes. This may introduce bias and limit the generalizability of findings to broader, real-world populations (Figure 1).^13^, ^14^, ^15^, ^16^, ^17^

The HONEST (coHOrte fraNcaise des dEfibrillateurs Sous cuTanés) cohort was designed to generate objective, unbiased, real-world data on a large population of S-ICD recipients, supported by rigorous, independent adjudication of clinical events. Initiated in 2018, the study benefits from the fact that the S-ICD is produced by a single manufacturer, allowing comprehensive, nationwide data collection. In addition, remote monitoring enables close follow-up of device function and clinical outcomes, ensuring consistent data quality throughout the study.

## Methods

### Design

The HONEST cohort is a French nationwide, single-arm, observational ongoing study that enrolled all patients who underwent implantation of an S-ICD (EMBLEM, Boston Scientific) between October 2012 (date of the first implantation in France) and December 2019 (Central Illustration). The study includes all 150 French Hospitals accredited to perform S-ICD implantations (Supplemental Table 1).

**Central Illustration:**
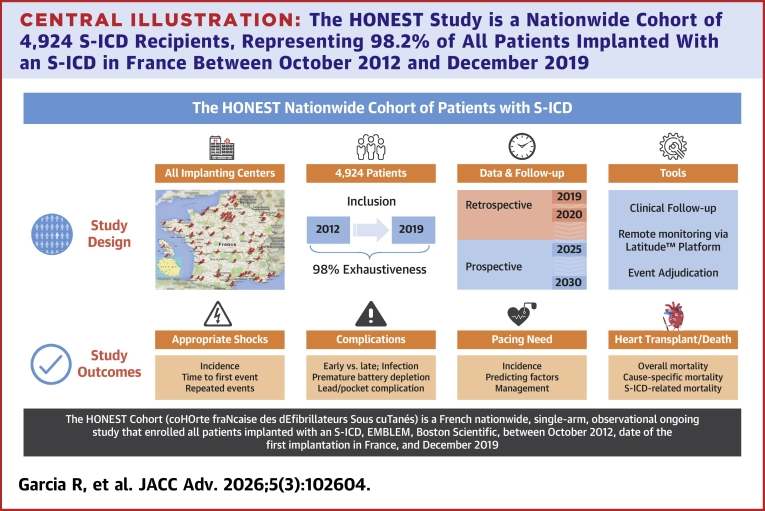
**The HONEST Study is a Nationwide Cohort of 4,924 S-ICD Recipients, Representing 98.2% of All Patients Implanted With an S-ICD in France Between October 2012 and December 2019** The cohort will provide comprehensive long-term evidence on outcomes associated with S-ICD therapy, thereby informing its safety, effectiveness, and real-world clinical utility. Beyond describing outcomes, HONEST also constitutes a robust research platform to support focused analyses and to refine contemporary approaches to sudden cardiac death prevention. HONEST = coHOrte fraNcaise des dEfibrillateurs Sous cuTanés; other abbreviation as in Figure 1.

Initiated in 2018 in collaboration with the French Society of Cardiology and part of the DAI-PP (Défibrillateur Automatique Implantable-Prévention Primaire) Research Network research program, the study was approved by Commission Nationale de l'Informatique et des Libertés, the French Data Protection Authority, under registration number CNIL2217196. It adheres to the principles of the Declaration of Helsinki and is registered at ClinicalTrials.gov (NCT05302115).

### Inclusion criteria

Patients were eligible for inclusion in the HONEST cohort if they met the following criteria:

1. Underwent implantation of an S-ICD (EMBLEM, Boston Scientific) in France between October 2012 and December 2019;
2. Provided informed consent to participate in the HONEST study, in accordance with the French MR-004 reference methodology for observational research involving human subjects.

### Exclusion criteria

The only exclusion criterion was refusal to provide informed consent in accordance with the MR-004 reference methodology.

### Study protocol

#### Inclusion and clinical follow-up

All patients who underwent S-ICD implantation in France since the first procedure in October 2012 were invited to participate in the HONEST study and to be continuously monitored via the Latitude remote monitoring system (Boston Scientific).

**Figure 2:**
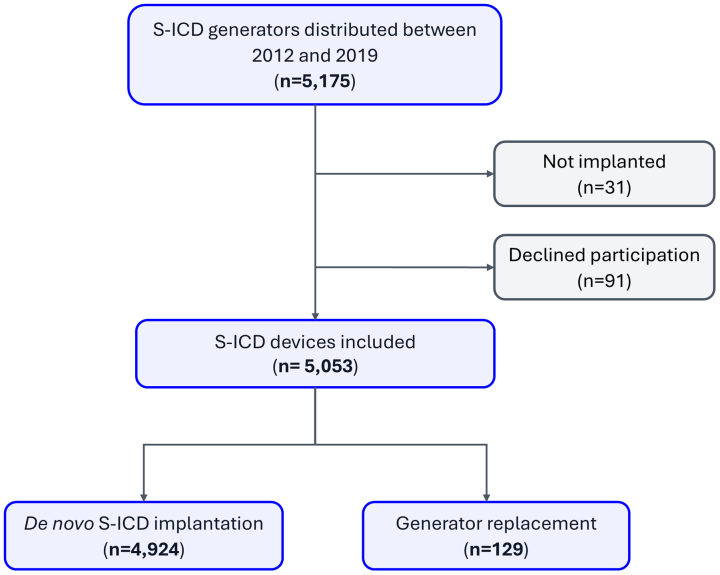
**Study Flowchart** The serial numbers corresponding to all S-ICD devices distributed in France from October 1, 2012, to December 31, 2019, were collected. Among 5,175 devices identified, 31 generators had been distributed to participating centers by the manufacturer but had not yet been implanted as of December 31, 2019, and 91 patients declined to participate. The final study population included 4,924 patients with de novo S-ICD implantation and 129 generator replacements, representing 98.2% of all patients implanted with an S-ICD in France during the study period. Abbreviation as in Figure 1.

Boston Scientific, the sole manufacturer of the S-ICD, provided a comprehensive list of serial numbers corresponding to all S-ICD devices distributed in France from October 1, 2012, to December 31, 2019, categorized by implanting center. These serial numbers were shared with all 150 accredited S-ICD implantation centers to allow patient identification and inclusion. During the study period (October 1, 2012 to December 31, 2019), a total of 5,175 S-ICD devices were distributed in France. Of these, 31 generators had been distributed to participating centers by the manufacturer but had not yet been implanted as of December 31, 2019, leaving 5,144 implanted S-ICDs. Among implanted devices, 91 patients declined participation. The HONEST cohort therefore includes 5,053 implanted S-ICD procedures, comprising 4,924 de novo implantations and 129 generator replacements. Overall, this corresponds to the capture of 98.2% of all S-ICD implantations performed in France during the study period (Figure 2). Participating centers included 39 university hospitals, 60 nonuniversity teaching hospitals, and 51 private institutions.

Patient data, including baseline characteristics, procedural details, and follow-up events, were retrospectively collected in 2019/2020.^18^ A prospective follow-up phase began in 2020 and is planned to continue annually for 10 years.

A prespecified electronic case report form was used to collect the following data: demographic variables, indication for ICD therapy (primary vs secondary prevention), underlying heart disease (diagnosis and specific characteristics), left ventricular ejection fraction, procedural data (including anesthesia modalities, S-ICD generator model [EMBLEM model 1010, A209 or A219], use of defibrillation testing, need of an additional pacing system, among others), and follow-up data, including the use of remote monitoring (Latitude, Boston Scientific) and the occurrence and exact timing of clinical events. These events included device-related complications, appropriate or inappropriate therapies, reinterventions, heart transplantation, and death. Moreover, patients’ genetic findings, along with raw intracardiac electrograms data recorded during electric shocks, are systematically collected. A complete list of collected outcome events is provided in Supplemental Table 2.

#### Study endpoints

The primary study outcomes at 5 and 10 years include 1) the cumulative incidence of appropriate shocks; 2) device-related complications, including inappropriate therapies, infection, lead dysfunction, and manufacturer-related recalls; 3) all-cause and cause-specific mortalities; and 4) requirement for pacing during follow-up.

An appropriate S-ICD shock was defined as any shock delivered for a ventricular arrhythmia with a ventricular rate exceeding the programmed therapy zone. Inappropriate shocks were defined as those triggered by nonventricular arrhythmias, T-wave oversensing, myocardial signals, or electromagnetic interference. Notably, shocks resulting from double counting of T-wave oversensing during slow ventricular tachycardia below the programmed therapy threshold was defined as inappropriate shock therapy. Surgical reintervention was defined as any procedure performed after initial implantation due to device-related complications. Local complications included premature battery depletion or pocket-related issues such as hematoma, localized or systemic infection, poor wound healing, and lead dislodgment. Early complications were defined as adverse events occurring within the first 30 days following S-ICD implantation. Late complications were defined as adverse events occurring beyond 30 days after S-ICD implantation.

### Data management and study organization

Study data were centrally collected and managed using REDCap electronic data capture tools hosted at the Paris Cardiovascular Research Center (U 970, European Georges Pompidou Hospital, Paris, France). Specific REDCap modules were created for each work package to streamline data entry and review.

A steering and scientific committee oversaw study conduct, data quality, and progress (Supplemental Table 3). Participating sites entered S-ICD shocks and device- or procedure-related complications into a centralized database. In parallel, an independent external research assistant reviewed source documentation from electronic health records and S-ICD interrogations, including stored electrograms, to verify, harmonize, and standardize site-reported data across centers. A dedicated adjudication committee conducted centralized reviews of key clinical endpoints, including cause of death (with specific focus on SCD and S-ICD–related deaths), device infections, lead-related complications, and shock episodes. Electrograms were systematically retrieved via remote monitoring. Among all recorded shocks, electrograms were independently reviewed in a blinded manner by 2 expert electrophysiologists in 81% to classify shocks as appropriate or inappropriate and determine the underlying rhythm mechanism. Any discordance between adjudicators or between central adjudication and site assessment was resolved by discussion and final determination during annual consensus meetings. To minimize loss to follow-up, a systematic process was implemented across all 150 participating centers to ensure completeness of longitudinal data. Notably, the study collects all events, not just the first occurrence, for outcomes such as appropriate therapies, enabling a comprehensive assessment of event burden over time.^19^ Finally, vital status is comprehensively ascertained through systematic cross-referencing with the National Institute of Statistics and Economic Studies (INSEE, France) database.

The HONEST study was initiated and is coordinated by the French Institute of Health and Medical Research (INSERM Unit 970) and the French Society of Cardiology. This investigator-initiated study is fully independent and received no industry funding. Of note, Boston Scientific had no involvement in the study design, data collection, analysis, interpretation, or the preparation of this report. The authors retain full responsibility for the study design, data analysis, manuscript preparation, and its final content.

### Statistical considerations

Categorical data are summarized as numbers and percentages, while continuous data are presented as mean values with SDs or as median values with IQRs, as appropriate. Cumulative incidence curves will be generated with the use of the Aalen–Johansen estimator, which accounts for competing events (eg, cardiovascular and noncardiovascular death). For noncompeting events, Kaplan-Meier curves will be used. Since the risk of events is not constant over time, cumulative incidence will be presented instead of incidence rates. For repeated events (eg, appropriate and inappropriate shocks and complications), cumulative hazard rate will be provided using the Nelson-Aalen estimator.

For multivariate analysis, a Cox proportional hazards model will be employed, using the Breslow method to handle tied event times. Time until events is recorded in days to minimize ties. A frailty term will be included to account for variability between trial sites, with center effects embedded as random to capture differences in clinical practices and center experience. For competing events (eg cause-specific deaths), both cause-specific hazard rate and subdistribution hazard rates will be calculated, with the Fine and Gray model used for sensitivity analyses. Prespecified predictors will be 1) patient characteristics (eg, age, sex, body mass index, prevention type [primary vs secondary], underlying heart disease category, left ventricular ejection fraction, prior ICD); 2) procedural/device characteristics (eg, incision technique [2-incision vs 3-incision], generator position [intermuscular vs subcutaneous], anesthesia type, defibrillation testing [performed vs not], device generation/type, SmartPass filter status); and 3) calendar time/center factors (eg, implant year/era, implanting center volume), when available.

For the analysis of appropriate and inappropriate shocks, the Anderson and Gill model will be applied, incorporating a robust sandwich variance estimator to correct for correlations between recurrent events within the same subject. Shocks occurring within the same episode (cluster) will be counted as a single shock. As a sensitivity analysis, a negative binomial model will be used to account for all shock episodes. For complications and complications requiring intervention, the Wei, Lin and Weissfeld model will be employed to account for repeated events of different nature, identifying factors associated with increased or reduced risks. The center effect will be examined as a fixed effect, categorized by centers with S-ICD implantation volumes greater or <100.

Follow-up will be censored in cases of loss to follow-up, device removal, death, or at the study’s end. The proportional hazards assumption will be assessed using statistical tests and graphical diagnostics based on scaled Schoenfeld residuals. Missing data will be described and handled using multiple imputation strategies. Clinically relevant variables and variables known to be outcome-related from existing literature will be included in the models.

Adjusted subgroup comparisons will be performed using inverse probability weighting based on propensity scores derived from logistic regression models incorporating age, body mass index, indication (primary vs secondary prevention), underlying heart disease, left ventricular ejection fraction, concomitant pacing system, prior ICD, anesthesia type, number of incisions, generator position, generator generation/type, and use of the SmartPass filter. Covariate balance before and after weighting will be evaluated using standardized mean differences, with standardized mean differences <0.10 indicating adequate balance, and will be visualized using Love plots.

To account for potential temporal confounding, outcomes will be analyzed stratified by calendar period and/or device generation, enabling interpretation of event rates in the context of evolving technology and clinical practice.

To quantify the precision afforded by the cohort size, we prespecified expected 95% CI widths for adverse event proportions in the full cohort and calculated illustrative minimum detectable effect sizes for two-group comparisons at 80% and 90% power (two-sided α = 0.05). For time-to-event endpoints, detectable HRs were estimated as a function of the expected number of observed events using the Schoenfeld approximation. These calculations are summarized in Supplemental Tables 4 to 6.

A 2-sided *P* value <0.05 will be considered statistically significant without alpha correction. All data are analyzed at French Institute of Health and Medical Research, Unit 970, Paris Cardiovascular Research Center, Paris, using the *R* software (R Project for Statistical Computing).

## Preliminary results

Preliminary retrospective data from the HONEST cohort were presented during a Late-Breaking Science session at the 2022 European Society of Cardiology Congress. A separate focused analysis comparing patients with and without congenital heart disease was also published.^18^ Additional HONEST analyses have evaluated the long-term impact of defibrillation testing performed at the time of S-ICD implantation,^20^ as well as outcomes following S-ICD implantation after complications related to a prior transvenous ICD.^21^

Of the 4,924 patients included in the cohort, the majority were male (n = 3,776; 76.7%), with a mean age of 49.9 years.^22^ The indication for implantation was primary prevention in 3,119 patients (63.3%). Structural heart diseases are present in 3,851 patients (78.2%), with the most common etiologies being coronary artery disease (n = 2,026, 52.6%) and hypertrophic cardiomyopathy (n = 497, 12.9%). Among patients with electrical heart diseases, Brugada syndrome was the most frequently observed condition (n = 473, 44.5%). General anesthesia was used in 3,883 patients (78.9%), and intraoperative defibrillation was performed in 4,065 cases (82.6%).

## Discussion

**Figure 3:**
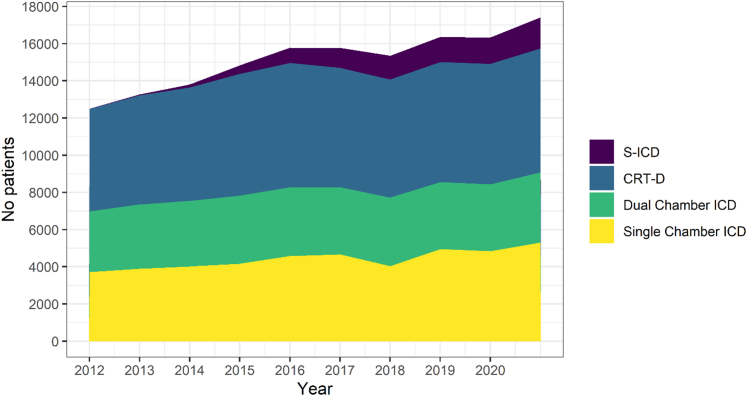
**Time-Trend Evolution of ICD Implantations in France** Absolute numbers of S-ICD implanted every year between 2012 and 2019. Overall, the total number of patients increased over time. The S-ICD uptake appears to have complemented rather than replaced transvenous ICD practice patterns over the same time frame. CRT-D = cardiac resynchronization therapy with defibrillator; ICD = implantable cardioverter-defibrillator; other abbreviation as in Figure 1.

At its inception, the S-ICD was anticipated to replace single-chamber transvenous ICDs in a substantial proportion of patients. However, despite capturing approximately 12% of the ICD market and exceeding 1,200 implantations annually in France (Figure 3), the volume of transvenous ICD procedures, particularly single-chamber devices, has remained stable since the introduction of the S-ICD.

Initially, the S-ICD was predominantly used in highly selected populations, such as relatively young patients with channelopathies or congenital heart disease.^23^^,^^24^ Over time, its use has broadened to include most patients with an ICD indication and no pacing requirement at the time of implantation.^25^ Meanwhile, the clinical landscape of ICD recipients has evolved considerably. Advances in heart failure pharmacotherapy have led to a reduction in the proportion of patients with significantly reduced ejection fraction, although the impact on SCD incidence remains uncertain.^26^ Additionally, catheter ablation has emerged as a cornerstone therapy for ventricular arrhythmias, as emphasized in recent guidelines, offering potential reductions in arrhythmia burden and mortality in selected populations.^27^, ^28^, ^29^

The HONEST cohort provides a unique opportunity to evaluate S-ICD utilization and outcomes comprehensively. Leveraging the fact that a single manufacturer produces all S-ICDs, the study achieved near-exhaustive inclusion of patients (98.2%) implanted with this device in France between 2012 and 2019. This enables robust analysis of clinical endpoints in the overall population as well as in key subgroups, including women, older adults, and patients with specific cardiac conditions. Ultimately, HONEST is positioned to improve our understanding of how the S-ICD can be optimized for the prevention of SCD.

The extensive database of arrhythmia events gathered in this study also offers a valuable resource for further research. Detailed analyses of both appropriate and inappropriate shocks could yield new insights into the initiation and maintenance of ventricular arrhythmias. Moreover, signal analysis of electrogram tracings may inform future improvements in arrhythmia prediction and detection algorithms as well as therapy delivery.^30^

To date, available data on S-ICDs have been consistently reassuring regarding safety and efficacy. Nevertheless, concerns persist regarding recalls, inappropriate shocks, and the need to upgrade to transvenous systems.^31^, ^32^, ^33^ The HONEST study, as an independent and comprehensive registry, will provide real-world data on device-related complications, including the incidence and mechanisms of inappropriate therapies and system malfunctions. It will also offer valuable insights into pacing requirements over time, contributing to improved patient selection and long-term management strategies.

Beyond its descriptive power, the HONEST cohort offers a unique opportunity to explore clinically relevant hypotheses that remain unanswered in the current literature. For example, the data set allows for evaluating whether the performance of defibrillation testing at the time of S-ICD implantation is associated with improved long-term outcomes, such as reduced arrhythmic mortality and/or fewer ineffective therapies. Additionally, anesthesia modality, recorded in detail across the entire cohort, may influence the risk of acute procedural complications or late lead migration, and warrants further investigation regarding its impact on overall device efficacy and patient safety. The large sample size and broad demographic spectrum also enable the analysis of sex-based differences in S-ICD outcomes, particularly relevant for a device implanted in the left pectoral region. Whether anatomical or physiological differences contribute to varying rates of inappropriate shocks, pocket complications, or sensing issues between men and women remains largely unknown and could be elucidated through this data set.

The study has several notable strengths. First, it represents one of the largest cohorts of S-ICD recipients to date, encompassing nearly 5,000 consecutive patients over a 7-year period. The sample size allows for precise estimates of adverse event rates, including appropriate and inappropriate shocks, and enables subgroup analyses, notably in patients with congenital heart disease and channelopathies. Second, the vast majority of patients are followed via remote monitoring, ensuring comprehensive electrogram capture and independent adjudication of arrhythmia events. Third, the HONEST cohort will provide long-term and extended follow-up data, up to 10 years, which is currently lacking in the literature, as most published studies are limited to 5 years of follow-up.^13^

Nevertheless, the study has limitations. Initial data collection was retrospective, although yearly prospective follow-up has been implemented since 2020. Verification of procedural and follow-up data in nearly all patients mitigates some of the inherent limitations of retrospective design. The inclusion period (2012-2019) reflects the earlier phase of S-ICD adoption. Nevertheless, this almost exhaustive nationwide cohort will enable a reliable evaluation of long-term outcomes after S-ICD implantation. Moreover, the absence of a comparator group (eg, patients with transvenous ICDs) limits the ability to directly assess the relative effectiveness of S-ICDs in preventing SCD.

## Conclusions

To the best of our knowledge, the HONEST study represents the largest real-world nationwide cohort of S-ICD recipients reported to date. It will generate comprehensive, long-term data on outcomes associated with S-ICD therapy, offering critical insight into its safety, efficacy, and clinical utility. Beyond its immediate clinical impact, the HONEST cohort also serves as a unique research platform for advancing the understanding and optimization of SCD prevention strategies.

## Funding support and author disclosures

This study is supported by the 10.13039/501100005630French Society of Cardiology, the 10.13039/501100003100French Federation of Cardiology, and the National Institute for Health and Medical Research. Dr Anselme is a consultant for Boston Scientific, Medtronic, and Microport. Dr Boveda is a consultant for Medtronic, Boston Scientific, Microport, and Zoll. Dr Defaye is a consultant for Boston Scientific, Medtronic, and Abbott. Dr Garcia is a consultant for Boston Scientific, Medtronic, Biotronik, Abbott, and Microport. Dr Marijon is a consultant for Abbott, Boston Scientific, and Medtronic and has received research grants from 10.13039/100000046Abbott, 10.13039/501100005035Biotronik, 10.13039/100008497Boston Scientific, 10.13039/100004374Medtronic, and 10.13039/501100018918Microport. All other authors have reported that they have no relationships relevant to the contents of this paper to disclose. Boston Scientific, the manufacturer of the studied device, had no involvement in the study design, data collection, analysis, interpretation, or the preparation of this report.
