# Supplementary material for: The HONEST Cohort Study: Rationale and Design of a Nationwide Subcutaneous Implantable Cardioverter-Defibrillator Cohort
Source: JACC Adv. 2026 Mar 25;5(3):102604. doi: 10.1016/j.jacadv.2026.102604 (PMC13351997; doi:10.1016/j.jacadv.2026.102604)
Supplement: Supplementary data [file mmc1.pdf]

SUPPLEMENTARY DATA

The HONEST Study: Rationale and Design of a Nationwide Subcutaneous Implantable Cardioverter-Defibrillator Cohort

Supplementary Table 1: List of Participating Centers and Investigators

Hospital	Zip Code	First Name	Last Name
Centre Hospitalier Saint Quentin	02100	Alexandre	Doucy
Centre Hospitalier de Moulins Yzeure	03000	Hassan	Barake
Centre Hospitalier de Moulins Yzeure	03000	Aziz	Kneizeh
CHU Nice - Nice	06000	Didier	Scarlatti
CHU Nice - Nice	06000	Fabien	Squara
CHU Nice - Nice	06000	Sithikun	Bun
Centre Arnault Tzanck - St Laurent du Var	06700	Philippe	Durand
Clinique Saint Georges - Nice	06000	Lara	Dabiri
Clinique Saint Georges - Nice	06000	Philippe	Ricard
Centre Hospitalier de Cannes	06400	Laurent	Liprandi
Centre Hospitalier de Cannes	06400	Garret	Gauthier
Centre Hospitalier d'Antibes - Juan Les Pins	06600	Folco	Frattini
Centre Hospitalier d'Antibes - Juan Les Pins	06600	Yann	Dagher hayeck
Centre Hospitalier de Troyes	10000	Alaa	Al Amoura
Centre Hospitalier de Troyes	10000	Bruno	Maillier
Centre Hospitalier de Carcassonne	11000	Denis	Gaty
Centre Hospitalier Rodez	12000	Lilian	Marty
Centre Hospitalier Rodez	12000	Mohammed	Benkaci Ali
Hôpital de la Timone - CHU Marseille	13385	Jean Claude	Deharo
Hôpital de la Timone - CHU Marseille	13385	Jérôme	Hourdain
Hôpital de la Timone - CHU Marseille	13385	Baptiste	Maille
Hôpital Nord - CHU Marseille	13915	Mickael	Peyrol
Hôpital Nord - CHU Marseille	13915	Jérémie	Barraud
Hôpital Nord - CHU Marseille	13915	Florian	Baptiste
Centre Hospitalier d' Aix-En-Provence	13100	Jerôme	Taieb
Centre Hospitalier d' Aix-En-Provence	13100	Jerôme	Bouet
Hôpital Privé Marseille - Beauregard	13012	Gilles	Macaluso
Hôpital privé Clairval - Marseille	13009	Alexis	Mechulan
Hôpital privé Clairval - Marseille	13009	Sébastien	Prevot
Hôpital privé Clairval - Marseille	13009	Ahmed	Bouharaoua
Hôpital Saint Joseph - Marseille	13008	Edouard	Gitenay
Hôpital Saint Joseph - Marseille	13008	Clément	Bars
Hôpital Saint Joseph - Marseille	13008	Julien	Seitz
CHU de Caen - Caen	14000	Laure	Champ Rigot
CHU de Caen - Caen	14000	Pierre	Ollitrault

CHU de Caen - Caen	14000	Paul	Milliez
CHU de Caen - Caen	14000	Arnaud	Pellissier
Hôpital privé Saint-Martin - Caen	14000	Alain	Lebon
Hôpital privé Saint-Martin - Caen	14000	Sophie	Gomes
Centre Hospitalier Henri Mondor - Aurillac	15002	Pamela	Damiano
Centre Hospitalier de La Rochelle	17000	Antoine	Milhem
Centre Hospitalier de La Rochelle	17000	Cécile	Duplantier
Centre Hospitalier Saintonge - Saintes	17100	Cyril	Goujeau
Centre Hospitalier Jacques Cœur - Bourges	18000	Isabelle	Heurtebise
Centre Hospitalier de Bastia - Bastia	20200	Lila	Khris
CHU Dijon Bourgogne - Dijon	21000	Charles	Guenancia
CHU Dijon Bourgogne - Dijon	21000	Géraldine	Bertaux
CHU Dijon Bourgogne - Dijon	21000	Gabriel	Laurent
CHU Dijon Bourgogne - Dijon	21000	Audrey	Sagnard
CHU Dijon Bourgogne - Dijon	21000	Marie	Fichot
Centre Hospitalier de Saint Brieuc	22000	Sylvain	Bodi
Centre Hospitalier de Saint Brieuc	22000	Anne	Quentin
Centre Hospitalier de Périgueux	24000	Jean	Litalien
Centre Hospitalier de Périgueux	24000	Mathieu	Courtheix
Centre Hospitalier de Périgueux	24000	Philippe	Jarnier
CHU Besançon	25000	Marc	Badoz
CHU Besançon	25000	Baptiste	Favoulet
CHU Besançon	25000	Guillaume	Serzian
Clinique Saint Vincent - Besançon	25000	Hugues	Zimmermann
Centre Hospitalier de Valence	26000	Aurélien	Miralles
Centre Hospitalier de Valence	26000	Marie	Pierre Chatel
Centre Hospitalier Eure-Seine - Évreux	27000	Éric	Ramiaramanana
Centre Hospitalier de Chartres	28000	Hervé	Gorka
Centre Hospitalier de Chartres	28000	Maria	Moldovan
Centre Hospitalier de Chartres	28000	Christophe	Laure
CHU de Brest	29200	Jacques	Mansourati
CHU de Brest	29200	Fawzi	Kerkouri
CHU de Brest	29200	Vincent	Mansourati
CHU de Brest	29200	Hugo	Hager
Hôpital Carémeau - CHU de Nîmes	30000	Pierre	Winum
Hôpital Carémeau - CHU de Nîmes	30000	Julien	Roux
Hôpital Privé Les Franciscaines - Nîmes	30000	Pénélope	Pujadas
CHU de Toulouse	31059	Pierre	Mondoly
CHU de Toulouse	31059	Philippe	Maury
CHU de Toulouse	31059	Guillaume	Domain
Clinique Pasteur - Toulouse	31000	Serge	Boveda
Clinique Pasteur - Toulouse	31000	Nicolas	Combes
Clinique Pasteur - Toulouse	31000	Stéphane	Combes
Clinique Pasteur - Toulouse	31000	Christèle	Cardin
Clinique Pasteur - Toulouse	31000	Jean-Paul	Albenque
Clinique Pasteur - Toulouse	31000	Olivier	Touboul
Clinique Pasteur - Toulouse	31000	Romain	Cassagneau
CHU de Bordeaux	33000	Pierre	Bordachar

CHU de Bordeaux	33000	Sylvain	Ploux
CHU de Bordeaux	33000	Frédéric	Sacher
CHU de Bordeaux	33000	Nicolas	Derval
CHU de Bordeaux	33000	Marc	Strik
Clinique Saint Augustin - Bordeaux	33000	Olivier	Cesari
Centre Hospitalier Libourne	33500	Rim	El Bouazzaoui
Centre Hospitalier Libourne	33500	Adlane	Zemmoura
Clinique du Parc - Castelnau le Lez - Montpellier	34170	Thien-Tri	Cung
Clinique du Millénaire - Montpellier	34000	Frederic	Cransac
Clinique du Millénaire - Montpellier	34000	Nicolas	Clementy
CHU de Montpellier	34000	Jean Luc	Pasquié
CHU de Montpellier	34000	Mathieu	Garnier
CHU Rennes - Hôpital Pontchaillou	35000	Nathalie	Behar
CHU Rennes - Hôpital Pontchaillou	35000	Christophe	Leclercq
CHU Rennes - Hôpital Pontchaillou	35000	Vincent	Galand
CHU Rennes - Hôpital Pontchaillou	35000	Raphael	Martins
CHU Rennes - Hôpital Pontchaillou	35000	Dominique	Pavin
Polyclinique Saint Laurent - Rennes	35000	Frédéric	Victor
CHU de Tours	37000	Bertrand	Pierre
CHU de Tours	37000	Laurent	Fauchier
CHU de Tours	37000	Arnaud	Bisson
Clinique Saint-Gatien - Tours	37000	Cyril	Zakine
Clinique Saint-Gatien - Tours	37000	Christophe	Loose
Clinique Saint-Gatien - Tours	37000	Akli	Otmani
CHU Grenoble - Alpes	38700	Pascal	Defaye
CHU Grenoble - Alpes	38700	Peggy	Jacon
CHU Grenoble - Alpes	38700	Adrien	Carabelli
CHU Grenoble - Alpes	38700	Sandrine	Venier
Clinique Belledonne - Grenoble	38400	Luc	Petit
Clinique Belledonne - Grenoble	38400	Xavier	Dreyfus
Centre Hospitalier de Mont de Marsan	40000	Corina	Moldovan
Centre Hospitalier Universitaire de Saint Etienne	42000	Antoine	Da Costa
Centre Hospitalier Universitaire de Saint Etienne	42000	Cécile	Romeyer-Bouchard
Centre Hospitalier Universitaire de Saint Etienne	42000	Jean-Bapt.	Guichard
Hôpital Privé de la Loire- Saint Etienne	42000	Jerôme	Thevenin
CHU de Nantes	44093	Vincent	Probst
CHU de Nantes	44093	Jean Baptiste	Gourraud
CHU de Nantes	44093	Marine	Arnaud
CHU de Nantes	44093	Damien	Minois
Hôpital privé du Confluent - Nantes	44000	Daniel	Gras
Clinique Oreliance - Orléans	45770	Cédric	Giraudeau
Clinique Oreliance - Orléans	45770	Radu	Moisei
Clinique Oreliance - Orléans	45770	René	Gabriel Huguet
Clinique Oreliance - Orléans	45770	Julien	Rischard
Clinique Oreliance - Orléans	45770	Soraya	Anys
CHU Angers	49000	Mouna	Ben Kilani
Clinique Esquirol Saint Hilaire - Agen	47000	Benjamin	Monteil

CHU Angers	49000	Sophie	Le Page
Clinique St-Joseph - Trélazé - Angers	49000	Frederic	Treguer
Clinique St-Joseph - Trélazé - Angers	49000	Michel	Merheb
CHU Reims	51100	Jean	Pierre Chabert
CHU Reims	51100	François	Lesaffre
CHU Reims	51100	Madeline	Espinosa
CHU Reims	51100	Nicolas	Luconi
Polyclinique Reims-Bezannes-Courlancy	51430	Thibault	Villemin
Polyclinique Reims-Bezannes-Courlancy	51430	Raphael	Sandras
Clinique Louis Pasteur Essey-lès-Nancy	54270	Karim	Bel Hadj
Clinique Louis Pasteur Essey-lès-Nancy	54270	Jerôme	Schwartz
Clinique Louis Pasteur Essey-lès-Nancy	54270	Arnaud	Olivier
Clinique Ambroise Paré - Nancy	54000	Daniel	Beurrier
Clinique Ambroise Paré - Nancy	54000	Pierre-Yves	Zinzius
CHU de Nancy	54000	Nicolas	Sadoul
CHU de Nancy	54000	Hugues	Blangy
CHU de Nancy	54000	Luc	Freysz
CHU de Nancy	54000	Christian	De Chillou
Centre hospitalier Bretagne Atlantique - Vannes	56000	Stéphane	Evain
Centre hospitalier Bretagne Atlantique - Vannes	56000	Éric	Rendu
Groupe Hospitalier Bretagne Sud - Lorient	56100	Pierre	Khattar
CHR Mercy - Metz	57000	Aude	Zanutto
CHR Mercy - Metz	57000	Marc	Mielczarek
CHR Mercy - Metz	57000	Julien	Bertrand
CHR Mercy - Metz	57000	Mathieu	Becker
Clinique Claude Bernard - Metz	57000	Pierre	Houriez
Hôpital Saint Philibert - Lille - GHICL	59160	Yves	Guyomard
Hôpital Saint Philibert - Lille - GHICL	59160	Aymeric	Menet
Polyclinique Vauban - Valencienne	59300	Olivier	Brimont
Centre Hospitalier de Roubaix	59100	Karine	Bauley
Centre Hospitalier de Roubaix	59100	Stéphane	Dennetiere
CHU de Lille	59037	Christelle	Marquié
CHU de Lille	59037	Charlotte	Potelle
CHU de Lille	59037	Sandro	Ninni
CHU de Lille	59037	Didier	Klug
Centre Hospitalier de Valenciennes	59300	Romain	Sellier
Centre Hospitalier de Valenciennes	59300	Laura	Forelle
Hôpital privé Le Bois - Lille Métropole	59000	Frédérique	Mizon-Gérard
Hôpital privé Le Bois - Lille Métropole	59000	Arthur	Vaksmann
Centre Hospitalier de Compiègne	60200	Frederic	Elmkies
Centre Hospitalier de Compiègne	60200	Thierry	Zerah
Centre Hospitalier de Boulogne sur Mer	62200	Éric	Verbrugge
Hôpital privé Bois Bernard - Lens	62300	Aurélie	Guiot
Hôpital privé Bois Bernard - Lens	62300	Marc	Poueymidanette
Centre de la Côte d'Opale - Boulogne Sur Mer	62200	Émilie	Marcant
Centre Hospitalier de Lens	62300	Claire	Vannesson
Centre Hospitalier de Lens	62300	Thibault	Hus
CHU Clermont- Ferrand	63003	Romain	Eschalier

CHU Clermont- Ferrand	63003	Frederic	Jean
CHU Clermont- Ferrand	63003	Grégoire	Massoullié
Pôle Santé République - Clermont- Ferrand	63100	Yannick	Saludas
Pôle Santé République - Clermont- Ferrand	63100	François	Philippot
Pôle Santé République - Clermont- Ferrand	63100	Antoine	Roux
Centre Hospitalier de Pau	64000	Maxime	De Guillebon
Centre Hospitalier de Pau	64000	Hugues	Bader
Centre Hospitalier de Pau	64000	Prune	Gaillard
Clinique Cardiologique D'Aressy	64320	Philippe	Couderc
Clinique Cardiologique D'Aressy	64320	Aurélien	Hebrard
GCS Cardiologie - Bayonne	64100	Nicolas	Klotz
GCS Cardiologie - Bayonne	64100	Julien	Laborderie
GCS Cardiologie - Bayonne	64100	Michel	Lerecouvreux
Centre Hospitalier de Bigorre - Tarbes	65000	Christian	Demasles
Centre Hospitalier de Bigorre - Tarbes	65000	Sorin	Pripon
Polyclinique de l'Ormeau - Tarbes	65000	Michel	Voglimacci
Polyclinique de l'Ormeau - Tarbes	65000	Dominique	Celse
Clinique Saint Pierre - Perpignan	66000	Philippe	Lagrange
Clinique Saint Pierre - Perpignan	66000	Ziad	khoueiry
Centre Hospitalier de Perpignan	66000	Pierre	Sultan
Centre Hospitalier de Perpignan	66000	Georges	Nadji
Centre Hospitalier de Haguenau (Est France)	67500	Mathieu	Steinbach
Centre Hospitalier de Haguenau (Est France)	67500	Sébastien	Bufflerin
Clinique de l'Orangerie - Strasbourg	67000	Michel	Chauvin
CHU de Strasbourg	67000	Alexandre	Schatz
CHU de Strasbourg	67000	Laurence	Jesel
GHCA - Colmar	68000	Sophie	Pynn
GHCA - Colmar	68000	Sandrine	Bellmont
Centre Hospitalier - Mulhouse - GHRMSA	68100	Jacques	Levy
Centre Hospitalier - Mulhouse - GHRMSA	68100	Ronan	Le Bouar
Centre Hospitalier - Mulhouse - GHRMSA	68100	Serban	Schiau
Centre Hospitalier - Mulhouse - GHRMSA	68100	Lucien	Diene
Hospices Civils de Lyon - Hôpital Louis Pradel	69500	Francis	Bessiere
Hospices Civils de Lyon - Hôpital Louis Pradel	69500	Arnaud	Dulac
Hospices Civils de Lyon - Hôpital Louis Pradel	69500	Philippe	Chevalier
Hospices Civils de Lyon - Hôpital Louis Pradel	69500	Kevin	Gardey
Clinique de l'Infirmerie Protestante de Lyon	69300	Cyril	Durand
Clinique de l'Infirmerie Protestante de Lyon	69300	Alexis	Durand Dubief
Clinique de l'Infirmerie Protestante de Lyon	69300	Hugo	Brahic
Clinique de l'Infirmerie Protestante de Lyon	69300	Hervé	Poty
Centre Hospitalier Saint Joseph Saint Luc - Lyon	69007	Benjamin	Gal
Centre Hospitalier Saint Joseph Saint Luc - Lyon	69007	Julien	Pineau
Centre Hospitalier Saint Joseph Saint Luc - Lyon	69007	Samuel	Chauveau
Clinique du Tonkin - Lyon - Villeurbane	69100	Olivier	Garrier
Polyclinique Lyon-Nord - Rillieux	69140	Michael	Attali
Hôpital de la Croix-Rousse - Lyon	69004	Samir	Fareh
Hôpital de la Croix-Rousse - Lyon	69004	Mathieu	Montoy
Hôpital de la Croix-Rousse - Lyon	69004	Pierre	Lantelme

Hôpital de la Croix-Rousse - Lyon	69004	Paul	Charles
Centre Hospitalier Chalon sur Saône	71100	Cédric	Nguyen
Centre Hospitalier du Mans	72037	Mathieu	Amelot
Pôle Santé Sud - Le Mans	72000	Philippe	Poret
Pôle Santé Sud - Le Mans	72000	Jean Chris.	Amirault
Centre Hospitalier de Chambéry	73000	Raoul	Bacquelin
Centre Hospitalier Annecy Genevois	74000	Pierre	Frey
Centre Hospitalier Annecy Genevois	74000	Didier	Irles
Centre Hospitalier Annecy Genevois	74000	Antoine	Dompnier
Centre Hospitalier Annecy Genevois	74000	Chrystelle	Akret
Institut Mutualiste Montsouris	75014	Edouard	Siméon
Institut Mutualiste Montsouris	75014	Olivier	Villejoubert
Institut Mutualiste Montsouris	75014	Nicolas	Mignot
Institut Mutualiste Montsouris	75014	Pierre	Jorrot
Hôpital St. Joseph	75014	Yamina	Mouhoub
Hôpital St. Joseph	75014	Lionel	Ovart
Clinique Alleray Labrouste	75015	Jacky	Ollitrault
Clinique Alleray Labrouste	75015	Denis	Amet
Hôpital européen Georges Pompidou	75015	David	Perrot
Hôpital européen Georges Pompidou	75015	Émilie	Varlet
Hôpital européen Georges Pompidou	75015	Pierre	Baudinaud
Hôpital européen Georges Pompidou	75015	Thomas	Lavergne
Hôpital européen Georges Pompidou	75015	Xavier	Jouven
Hôpital européen Georges Pompidou	75015	Séverine	Philibert
Hôpital européen Georges Pompidou	75015	Pauline	Pinon
Hôpital européen Georges Pompidou	75015	Tej	Chalbia
Hôpital européen Georges Pompidou	75015	Victor	Waldmann
Hôpital européen Georges Pompidou	75015	Eloi	Marijon
Hôpital La Pitié Salpêtrière	75013	Nicolas	Badenco
Hôpital La Pitié Salpêtrière	75013	Estelle	Gandjbakhch
Hôpital La Pitié Salpêtrière	75013	Guillaume	Duthoit
Hôpital La Pitié Salpêtrière	75013	Mikael	Laredo
Hôpital La Pitié Salpêtrière	75013	Xavier	Waintraub
Hôpital Bichat	75018	Anne	Messali
Hôpital Bichat	75018	Antoine	Leenhardt
Hôpital Bichat	75018	Vincent	Algalarrondo
Hôpital Bichat	75018	Fabrice	Extramiana
Hôpital Necker	75015	Victor	Waldmann
Hôpital Necker	75015	Damien	Bonnet
CHU de Rouen	76000	Benedicte	Godin
CHU de Rouen	76000	Frederic	Anselme
CHU de Rouen	76000	Arnaud	Savouré
CHU de Rouen	76000	Corentin	Chaumont
Groupe Hospitalier du Havre	76600	Nathanael	Auquier
Groupe Hospitalier du Havre	76600	Popescu	Elena
Clinique Saint-Hilaire - Rouen	76000	Pierre	Le Franc
Clinique Saint-Hilaire - Rouen	76000	Fanny	Bouchinet
Clinique Les Fontaines - Melun	77000	Cyrus	Moini

Clinique Les Fontaines - Melun	77000	Audrey	Lefoulon
Grand Hôpital de l'Est Francilien - Marne-La-Vallée	77600	Mohamed	Belhameche
Grand Hôpital de l'Est Francilien - Marne-La-Vallée	77600	Sana	Sioua
Hôpital privé de Parly 2	78150	Abdeslam	Bouzeman
Hôpital privé de Parly 2	78150	Cathy	Bertrand
Hôpital privé de Parly 2	78150	Franck	Halimi
CHI de Poissy - Saint Germain en Laye	78100	Thomas	Chastre
CHI de Poissy - Saint Germain en Laye	78100	Khadija	Belkhir
CHI de Poissy - Saint Germain en Laye	78100	Raphael	Gdalia
CHI de Poissy - Saint Germain en Laye	78100	Jean-Pierre	Jabbour
CHI de Poissy - Saint Germain en Laye	78100	Denis	Amet
CHU Amiens	80054	Jean-Sylvain	Hermida
CHU Amiens	80054	Alexis	Hermida
CHU Amiens	80054	Akli	Otmani
CHU Amiens	80054	Maciej	Kubala
SAS Cardiologie et Urgences - Amiens	80000	Sarah	Traulle
SAS Cardiologie et Urgences - Amiens	80000	Denis	Raguin
Centre Hospitalier d'Albi	81000	Marie	Blaye-Felice
Centre Hospitalier d'Albi	81000	Philippe	Rumeau
Centre Hospitalier Intercommunal Castres-Mazamet	81100	Pascal	Chavernac
Centre Hospitalier Intercommunal Castres-Mazamet	81100	Marion	Pouche
Centre Hospitalier de Montauban	82000	Nouredine	El Hajjaji
Polyclinique Les Fleurs - Toulon	83000	Émilie	Bastard
Centre Hospitalier Toulon	83000	Isabelle	Lecardonnel
Centre Hospitalier Toulon	83000	Essia	Lakhal
Hôpital d'instruction des armées Sainte-Anne - Toulon	83000	Gilles	Cellarier
Hôpital d'instruction des armées Ste-Anne - Toulon	83000	Raphaël	Demoulin
Centre Hospitalier d'Avignon	84000	Olivier	Barthez
Centre Hospitalier d'Avignon	84000	Jean	Paul Faugier
Centre Hospitalier d'Avignon	84000	Saida	Cheggour
Clinique Rhône Durance - Avignon	84000	François	Xavier Hager
Clinique Rhône Durance - Avignon	84000	Frédéric	Ortuno
Centre Hospitalier Vendée - La Roche-sur-Yon	85000	Olivier	Billon
CHU de Poitiers	86000	Rodrigue	Garcia
CHU de Poitiers	86000	Bruno	Degand
CHU de Poitiers	86000	François	Le Gal
CHU de Limoges	87000	Benoit	Guy Moyat
Centre Hospitalier d’Auxerre	89000	François	Jourda
Centre Hospitalier d’Auxerre	89000	Stéphane	Mourot
Hôpital Nord Franche-Comté	90400	Renaud	Fouché
Institut Jacques Cartier - Massy	91300	Jerôme	Horvilleur
Institut Jacques Cartier - Massy	91300	Laurent	Fiorina
Institut Jacques Cartier - Massy	91300	Jerôme	Lacotte
Institut Jacques Cartier - Massy	91300	Fiorella	Salerno
Institut Jacques Cartier - Massy	91300	Salem	Younsi
Institut Jacques Cartier - Massy	91300	Mina	Ait Said
Institut Jacques Cartier - Massy	91300	Vladimir	Manenti

Centre Hospitalier Sud Francilien - Corbeil-Essonnes	91106	Mohanad	Mahfoud
Centre Hospitalier Sud Francilien - Corbeil-Essonnes	91106	Jacques	Monteau
Hôpital privé Claude Galien - Quincy-sous-Sénart	91480	Vladimir	Manenti
Hôpital privé Claude Galien - Quincy-sous-Sénart	91480	Dominique	Bleinc
Clinique Ambroise Paré - Neuilly-sur-Seine	92200	Christine	Alonso
Clinique Ambroise Paré - Neuilly-sur-Seine	92200	Arnaud	Lazarus
Clinique Ambroise Paré - Neuilly-sur-Seine	92200	Ghassan	Moubarak
Clinique Ambroise Paré - Neuilly-sur-Seine	92200	Olivier	Thomas
Clinique Ambroise Paré - Neuilly-sur-Seine	92200	Ardalan	Sharifzadehgan
Clinique Ambroise Paré - Neuilly-sur-Seine	92200	David	Perrot
Clinique Ambroise Paré - Neuilly-sur-Seine	92200	Alexandre	Zhao
Hôpital Antoine-Béclère - Clamart	92140	Christophe	Juin
Hôpital d'Instruction des Armées - Percy	92140	Vincent	Kanczuga
Hôpital d'Instruction des Armées - Percy	92140	Henri	Broustet
Hôpital Marie Lannelongue - Le Plessis Robinson	92350	Nicolas	Combes
Hôpital Marie Lannelongue - Le Plessis Robinson	92350	Alice	Maltret
Centre Cardiologique du Nord - Saint Denis	93200	Abdelhamid	Benounane
Centre Cardiologique du Nord - Saint Denis	93200	Xavier	Copie
Centre Cardiologique du Nord - Saint Denis	93200	Olivier	Piot
Groupe Hospitalier Le Raincy Montfermeil	93370	Walid	Amara
Groupe Hospitalier Le Raincy Montfermeil	93370	Vanessa	Abdou
Groupe Hospitalier Le Raincy Montfermeil	93370	Fabien	Monsel
Hôpital Henri Mondor - Créteil	94010	Nicolas	Lellouche
Hôpital Henri Mondor - Créteil	94010	Nathalie	Elbaz
Hôpital Henri Mondor - Créteil	94010	Ségolène	Rouffiac-Noel
Centre Hospitalier d'Argenteuil - Argenteuil	95100	Guillaume	Galidie
Centre Hospitalier René Dubos - Pontoise	95300	Dorian	Nitu
Centre Hospitalier Princesse Grace - Monaco	98000	Gabriel	Latcu
Centre Hospitalier Princesse Grace - Monaco	98000	Bogdan	Enache
Centre Cardio-Thoracique de Monaco- Monaco	98000	Nicolas	Hugues
Centre Hospitalier de Basse-Terre - Basse-Terre	97100	Isabelle	Lagrenade
CHU Fort-de-France - Fort-de-France	97200	Fabrice	Demoniere
CHU Fort-de-France - Fort-de-France	97200	Andréas	Müssigbrodt
CHU de la Réunion - Saint Pierre	97410	Olivier	Geoffroy
CHU de la Réunion - Saint Pierre	97410	Gaël	Clerici
CHU de la Réunion - Saint Pierre	97410	François	Wiart
Centre hospitalier de la Polynésie française - Papeete	98713	Bruno	Ulmer
Centre Hospitalier Territorial- Nouméa	98849	Guillaume	Kabalu
Centre Hospitalier Territorial- Nouméa	98849	Olivier	Axler

Supplementary Table 2. Outcome Events collected in HONEST Cohort study

**Appropriate shocks[1]**	Number of appropriate shocks, date of each appropriate shock
**Complications**	(1) Inappropriate shocks[2]: number of shocks, cause and date of each event<br>(2) Local complications:<br>- Infection: local or systemic infection, and date of occurrence<br>- Pocket hematoma with date of occurrence<br>- Poor wound healing[3] with the date of occurrence<br>- Other local complications as a free field to fill with their dates of occurrence<br>(3) Lead dysfunctions:<br>- Lead dislodgement with date of occurrence<br>- Other lead complications as a free field to fill with their dates of occurrence<br>(4) Early battery depletions4 or recalls: type and date of occurrence<br>(5) S-ICD related death: details about the circumstances of death with the date of occurrence<br>(6) Other complications: type of complications other than abovementioned with their date of occurrence
**Interventions[5]**	(1) Number of interventions<br>(2) Cause of intervention:<br>- S-ICD related complication: type of complication<br>- Pacing need: sinus dysfunction, AV block, CRT, ATP<br>- Generator change<br>- Other reason as a free field to fill<br>(3) Type of intervention:<br>- Intervention without device extraction<br>- Device extraction6 with new S-ICD implantation<br>- Definite extraction7 with epicardial/ transvenous ICD implantation<br>- Definite extraction without ICD implantation<br>(4) Date of each intervention during follow up
**Heart Transplantation**	Date of heart transplantation
**Death[8]**	(1) Date of death<br>(2) Cause of death:<br>- Cardiovascular: with specifying the principal reason of death<br>- Non-cardiovascular: with specifying the principal reason of death<br>- S-ICD-related: with details about the circumstances of death<br>- Unknown

[*] *Data were collected from 2 sources: 1) Telemonitoring of S-ICD providing details about shocks and battery status 2) Routine clinical follow up.*

[1] *An appropriate shock is defined as a shock delivered by the S-ICD for a ventricular arrythmia with a rate above the lower limit of the programmed therapy zone.*

[2] *An Inappropriate shock is defined as an S-ICD delivered shock not fulfilling the appropriate shock definition.*

[3] *A pour wound healing encompasses significant delayed wound healing, wound disunion without clear signs of infection or keloid scarring.*

[4] *An Early battery depletion is defined as a generator replacement prior to 60 months since implantation without shock deliveries or an unexpected decrease in absolute battery longevity greater than 25% within 12 months leading to generator change prior to 90 months.*

[5] *An intervention involves any requirement for invasive treatment, device revision or implantation of a pacing system.*

[6]A device extraction refers to a complete extraction of the -S-ICD, including both the generator and the lead.
[7]A definite extraction refers to the absence of subsequent implantation of another S-ICD following the complete extraction of the device.
[8]All patients, including those who had definitively undergone S-ICD removal, underwent a verification process within the National Institute of Statistics and Economic Studies (INSEE, France) database to ascertain and validate their vital status. Consequently, no competing risk is present regarding the estimation of outcomes events and deaths.
Abbreviations: S-ICD denotes Subcutaneous implantable cardioverter defibrillator, ICD implantable cardioverter defibrillator, AV atrioventricular, CRT cardiac resynchronization therapy and ATP anti-tachycardia therapy.

Supplementary Table 3 : Steering, Scientific and Adjudication HONEST Committees

Steering Committee Members

Name	Center
**Serge Boveda**	Clinique Pasteur, Toulouse
**Rodrigue Garcia**	Centre hospitalier universitaire de Poitiers
**Fawzi Kerkouri**	Centre hospitalier universitaire de Brest
**Eloi Marijon**	Hôpital européen Georges Pompidou, Paris
**David Perrot**	Hôpital européen Georges Pompidou, Paris

Scientific Committee Members

Name	Center
**Frédéric Anselme**	Centre hospitalier universitaire de Rouen
**Serge Boveda**	Clinique Pasteur, Toulouse
**Michel Chauvin**	Institut cardiovasculaire de Strasbourg
**Pascal Defaye**	Centre hospitalier universitaire de Grenoble
**Rodrigue Garcia**	Centre hospitalier universitaire de Poitiers
**Jérôme Hourdain**	Centre hospitalier universitaire de Marseille, hôpital de la Timone
**Christophe Leclercq**	Centre hospitalier universitaire de Rennes
**Christelle Marquié**	Centre hospitalier universitaire de Lille
**Eloi Marijon**	Hôpital européen Georges Pompidou, Paris
**Pierre Mondoly**	Centre hospitalier universitaire de Toulouse
**Vincent Probst**	Centre hospitalier universitaire de Nantes
**Nicolas Sadoul**	Centre hospitalier universitaire de Nancy

Adjudication Committee Members

Name	Center
**Frédéric Anselme**	Centre hospitalier universitaire de Rouen
**Nicolas Badenco**	La Pitié Salpétrière, Paris
**Pierre Bertrand**	Centre hospitalier universitaire de Tours
**Geraldine Bertaux**	Centre hospitalier universitaire de Dijon
**Hugues Blangy**	Centre hospitalier universitaire de Nancy
**Serge Boveda**	Clinique Pasteur, Toulouse
**Michel Chauvin**	Institut cardiovasculaire de Strasbourg
**Laure Champ-Rigot**	Centre hospitalier universitaire de Caen
**Estelle Gandjbakhch**	La Pitié Salpétrière, Paris
**Rodrigue Garcia**	Centre hospitalier universitaire de Poitiers
**Charles Guenancia**	Centre hospitalier universitaire de Dijon
**Jérôme Hourdain**	Centre hospitalier universitaire de Marseille, hôpital de la Timone
**Peggy Jacon**	Centre hospitalier universitaire de Grenoble
**Jacques Mansourati**	Centre hospitalier universitaire de Brest
**Christelle Marquié**	Centre hospitalier universitaire de Lille
**Eloi Marijon**	Hôpital européen Georges Pompidou, Paris
**Philippe Maury**	Centre hospitalier universitaire de Toulouse
**Pierre Mondoly**	Centre hospitalier universitaire de Toulouse

**Vincent Probst**	Centre hospitalier universitaire de Nantes
**Nicolas Sadoul**	Centre hospitalier universitaire de Nancy
**Jean Marc Sellal**	Centre hospitalier universitaire de Nancy

Supplementary Table 4 : Precision for an adverse event proportion in the full cohort (binomial; normal approximation*)

**Assumed adverse event proportion (p)**	**Expected 95% CI half-width**	**Expected total 95% CI width**
1%	±0.28 percentage points	0.56 percentage points
2%	±0.39 percentage points	0.78 percentage points
5%	±0.61 percentage points	1.22 percentage points
10%	±0.84 percentage points	1.68 percentage points

* 95% CI half-width approximated as $1.96\sqrt{p(1-p)/n}$. Exact (Clopper–Pearson) binomial CIs will be reported for rare adverse events

Supplementary Table 5 : Two-group AE proportion comparison: minimum detectable absolute difference (percentage points)* (Assuming baseline event rate p0 in the reference group)

**Baseline adverse event (p0)**	**Allocation**	**Min detectable Δ (80% power)**	**Min detectable Δ (90% power)**
2%	1:1	1.28 pp	1.51 pp
2%	1:3	1.40 pp	1.63 pp
5%	1:1	1.89 pp	2.21 pp
5%	1:3	2.11 pp	2.45 pp
10%	1:1	2.52 pp	2.94 pp
10%	1:3	2.85 pp	3.31 pp

*Approximate two-sample proportion test; values are illustrative and intended to contextualize precision/detectable effects for common subgroup splits (1:1 and 1:3).

Supplementary Table 6 : Time-to-event endpoints (Cox): detectable hazard ratio (HR) as a function of observed events (D)*

**Observed events (D)**	**Allocation**	**Detectable HR (80% power)**	**Detectable HR (90% power)**
150	1:1	1.58	1.70
150	1:3	1.70	1.84
250	1:1	1.43	1.51
250	1:3	1.51	1.61
400	1:1	1.32	1.38
400	1:3	1.38	1.45
600	1:1	1.26	1.30
600	1:3	1.30	1.36

* Schoenfeld approximation: $\log(HR) \approx (z_{1-\alpha/2} + z_{1-\beta})/\sqrt{D \cdot p(1-p)}$, where $p$is the allocation proportion. Final power depends on the observed event counts, censoring, and covariate adjustment.
