# Supplementary material for: The HONEST Cohort Study: Rationale and Design of a Nationwide Subcutaneous Implantable Cardioverter-Defibrillator Cohort
Source: JACC Adv. 2026 Mar 25;5(3):102604. doi: 10.1016/j.jacadv.2026.102604 (PMC13351997; doi:10.1016/j.jacadv.2026.102604)
Supplement: Supplementary file 2 [file mmc2.docx]

**List of Participating Centers**

| Department | Center | Investigators |
| --- | --- | --- |
| 2 | Centre Hospitalier Saint Quentin | Dr Alexandre Doucy |
| 3 | Centre Hospitalier de Moulins Yzeure | Dr Hassan Barake / Dr Aziz Kneizeh |
| 6 | CHU Nice - Nice | Dr Didier Scarlatti / Dr Fabien Squara  Dr Sok-Sithikun Bun |
| 6 | Centre Médico-Chirurgical Arnault Tzanck - St Laurent du Var | Dr Philippe Durand |
| 6 | Clinique Saint Georges - Nice | Dr Lara Dabiri / Dr Philippe Ricard |
| 6 | Centre Hospitalier de Cannes | Dr Laurent Liprandi / Dr Garret Gauthier |
| 6 | Centre Hospitalier d'Antibes - Juan Les Pins | Dr Folco Frattini / Dr Yann Dagher Hayeck |
| 10 | Centre Hospitalier de Troyes | Dr Alaa Al Amoura / Dr Bruno Maillier |
| 11 | Centre Hospitalier de Carcassonne | Dr Denis Gaty |
| 12 | Centre Hospitalier Rodez | Dr Lilian Marty / Dr Mohammed Benkaci Ali |
| 13 | Hôpital de la Timone - CHU Marseille | Dr Jean Claude Deharo / Dr Jérome Hourdain  Dr Baptiste Maille |
| 13 | Hôpital Nord - CHU Marseille | Dr Mickael Peyrol / Dr Jérémie Barraud  Dr Florian Baptiste |
| 13 | Centre Hospitalier d’Aix-En-Provence | Dr Jerome Taieb / Dr Jerome Bouet |
| 13 | Hôpital Privé Marseille - Beauregard | Dr Gilles Macaluso |
| 13 | Hôpital privé Clairval - Marseille | Dr Alexis Mechulan / Dr Sébastien Prevot  Dr Ahmed Bouharaoua |
| 13 | Hôpital Saint Joseph - Marseille | Dr Edouard Gitenay / Dr Clement Bars  Dr Julien Seitz |
| 14 | CHU de Caen - Caen | Dr Laure Champ Rigot / Dr Pierre Ollitrault  Dr Paul-Ursmar Milliez / Dr Arnaud Pellissier |
| 14 | Hôpital privé Saint-Martin - Caen | Dr Alain Lebon / Dr Sophie Gomes |
| 15 | Centre Hospitalier Henri Mondor - Aurillac | Dr Pamela Damiano |
| 17 | Centre Hospitalier de La Rochelle | Dr Antoine Milhem / Dr Cecile Duplantier |
| 17 | Centre Hospitalier Saintonge - Saintes | Dr Cyril Goujeau |
| 18 | Centre Hospitalier Jacques Cœur - Bourges | Dr Isabelle Heurtebise / Dr Vincent Ahfat |
| 20 | Centre Hospitalier de Bastia - Bastia | Dr Lila Khris |
| 21 | CHU Dijon Bourgogne - Dijon | Dr Charles Guenancia / Dr Géraldine Bertaux  Dr Gabriel Laurent/ Dr Audrey Sagnard  Dr Marie Fichot |
| 22 | Centre Hospitalier de Saint Brieuc | Dr Sylvain Bodi / Dr Anne Quentin  Dr Jonathan Lacaze Gadonneix |
| 24 | Centre Hospitalier de Périgueux | Dr Jean Litalien / Dr Mathieu Courtheix  Dr Philippe Jarnier |
| 25 | CHU Besançon | Dr Marc Badoz / Dr Baptiste Favoulet  Dr Guillaume Serzian |
| 25 | Clinique Saint Vincent - Besancon | Dr Hugues Zimmermann |
| 26 | Centre Hospitalier de Valence | Dr Aurélien Miralles / Dr Marie Pierre Chatel |
| 27 | Centre Hospitalier Eure-Seine - Evreux | Dr Eric Ramiaramanana |
| 28 | Centre Hospitalier de Chartres | Dr Hervé Gorka / Maria Moldovan  Dr Christophe Laure |
| 29 | Hôpital la Cavale Blanche - CHU de Brest | Dr Jacques Mansourati / Dr Fawzi Kerkouri  Dr Vincent Mansourati |
| 30 | Hôpital Carémeau - CHU de Nîmes | Dr Pierre Winum / Dr Julien Roux |
| 30 | Hôpital Privé Les Franciscaines - Nîmes | Dr Pénélope Pujadas |
| 31 | CHU de Toulouse | Dr Pierre Mondoly / Dr Philippe Maury  Dr Guillaume Domain |
| 31 | Clinique Pasteur - Toulouse | Dr Serge Boveda / Dr Nicolas Combes  Dr Stéphane Combes / Dr Christèle Cardin  Dr Jean-Paul Albenque / Dr Romain Cassagneau |
| 33 | CHU de Bordeaux | Dr Pierre Bordachar / Dr Sylvain Ploux  Dr Frédéric Sacher / Dr Nicolas Derval |
| 33 | Clinique Saint Augustin - Bordeaux | Dr Olivier Cesari |
| 33 | Centre Hospitalier Libourne | Dr Rim El Bouazzaoui / Dr Adlane Zemmoura |
| 34 | Clinique du Parc - Castelnau le Lez - Montpellier | Dr Thien-Tri Cung |
| 34 | Clinique du Millénaire - Montpellier | Dr Frederic Cransac / Dr Nicolas Clementy  Dr Pierre GALLAY / Dr Maxime PONS |
| 34 | CHU de Montpellier | Dr Jean Luc Pasquié/ Dr Mathieu Garnier |
| 35 | CHU Rennes - Hôpital Pontchaillou | Dr Nathalie Behar / Dr Christophe Leclercq  Dr Vincent Galand / Dr Raphael Martins  Dr Dominique Pavin |
| 35 | Polyclinique Saint Laurent - Rennes | Dr Frédéric Victor |
| 37 | CHU de Tours | Dr Bertrand Pierre / Dr Laurent Fauchier  Dr Arnaud Bisson |
| 37 | Clinique Saint-Gatien - Tours | Dr Cyril Zakine / Dr Christophe Loose  Dr Akli OTMANI |
| 38 | CHU Grenoble - Alpes | Dr Pascal Defaye / Dr Peggy Jacon /  Dr Adrien Carabelli / Dr Sandrine Venier |
| 38 | Clinique Belledonne - Grenoble | Dr Luc Petit / Dr Xavier Dreyfus |
| 40 | Centre Hospitalier de Mont de Marsan | Dr Corina Moldovan |
| 42 | Centre Hospitalier Universitaire de Saint Etienne | Dr Antoine Da Costa / Dr Cécile Romeyer-Bouchard/ Dr Jean-Baptiste Guichard |
| 42 | Hôpital Privé de la Loire- Saint Etienne | Dr Jerome Thevenin |
| 44 | CHU de Nantes | Dr Vincent Probst / Dr Jean Baptiste Gourraud  Dr Antoine Andorin / Dr Damien Minois |
| 44 | Hôpital privé du Confluent - Nantes | Dr Daniel Gras |
| 45 | Clinique Oreliance - Orléans | Dr Cédric Giraudeau / Dr Radu Moisei  Dr René Gabriel Huguet / Dr Julien Rischard  Dr Soraya Anys |
| 47 | Clinique Esquirol Saint Hilaire - Agen | Dr Benjamin Monteil |
| 49 | CHU Angers | Dr Sophie Le Page / Dr Mouna Ben Kilani  Dr Jean-Marc Dupuis |
| 49 | Clinique St-Joseph - Trélazé - Angers | Dr Frederic Treguer / Dr Michel Merheb |
| 51 | CHU Reims | Dr Jean Pierre Chabert / Dr Francois Lesaffre  Dr Madeline Espinosa / Dr Nicolas Luconi |
| 51 | Polyclinique Reims-Bezannes-Courlancy | Dr Thibault Villemin / Dr Raphael Sandras |
| 54 | Clinique Louis Pasteur Essey-lès-Nancy | Dr Karim Bel Hadj / Dr Jerome Schwartz  Dr Arnaud Olivier |
| 54 | Clinique Ambroise Paré - Nancy | Dr Daniel Beurrier / Dr Pierre-Yves Zinzius |
| 54 | CHU de Nancy | Dr Nicolas Sadoul / Dr Hugues Blangy  Dr Luc Freysz / Dr Christian De Chillou |
| 56 | Centre hospitalier Bretagne Atlantique - Vannes | Dr Stéphane Evain / Dr Eric Rendu |
| 56 | Groupe Hospitalier Bretagne Sud - Hôpital du Scorff - Lorient | Dr Pierre Khattar |
| 57 | CHR Mercy - Metz | Dr Aude Zanutto / Dr Marc Mielczarek  Dr Julien Bertrand / Dr Mathieu Becker |
| 57 | Clinique Claude Bernard - Metz | Dr Pierre Houriez |
| 59 | Hôpital Saint Philibert - Lille - GHICL | Dr Yves Guyomard / Dr Aymeric Menet |
| 59 | Polyclinique Vauban - Valencienne | Dr Olivier Brimont |
| 59 | Centre Hospitalier de Roubaix | Dr Karine Bauley / Dr Stephane Dennetiere |
| 59 | CHU de Lille | Dr Christelle Marquié / Dr Charlotte Potelle  Dr Didier Klug |
| 59 | Centre Hospitalier de Valenciennes | Dr Romain Sellier / Dr Laura Forelle |
| 59 | Hôpital privé Le Bois - Lille Metropole | Dr Frédérique Mizon-Gérard  Dr Arthur Vaksmann |
| 60 | Centre Hospitalier de Compiègne | Dr Frederic Elmkies / Dr Thierry Zerah |
| 62 | Centre Hospitalier de Boulogne sur Mer | Dr Eric Verbrugge |
| 62 | Hôpital privé Bois Bernard - Lens | Dr Aurélie Guiot / Dr Marc Poueymidanette |
| 62 | Centre Médical Chirurgical Obstétrical de la Côte d'Opale - Boulogne Sur Mer | Dr Emilie Marcant |
| 62 | Centre Hospitalier de Lens | Dr Claire Vanesson / Dr Thibault Hus |
| 63 | CHU Clermont- Ferrand | Dr Romain Eschalier / Dr Frederic Jean  Dr Grégoire Massoullié |
| 63 | Pôle Santé République - Clermont- Ferrand | Dr Yannick Saludas / Dr François Philippot  Dr Antoine Roux |
| 64 | Centre Hospitalier de Pau | Dr Maxime De Guillebon / Dr Hugues Bader  Dr Prune Gaillard |
| 64 | Clinique Cardiologique D'Aressy | Dr Philippe Couderc / Dr Aurelien Hebrard |
| 64 | GCS Cardiologie - Bayonne | Dr Nicolas Klotz / Dr Julien Laborderie  Dr Michel Lerecouvreux |
| 65 | Centre Hospitalier de Bigorre - Tarbes | Dr Christian Demasles / Dr Sorin Pripon |
| 65 | Polyclinique de l'Ormeau - Tarbes | Dr Michel Voglimacci / Dr Dominique Celse |
| 66 | Clinique Saint Pierre - Perpignan | Dr Philippe Lagrange / Dr Ziad khoueiry |
| 66 | Centre Hospitalier de Perpignan | Dr Pierre Sultan/ Dr Georges Nadji |
| 67 | Centre Hospitalier de Haguenau (Est France) | Dr Mathieu Steinbach / Dr Sebastien Bufflerin |
| 67 | Clinique de l'Orangerie - Strasbourg | Dr Michel Chauvin |
| 67 | CHU de Strasbourg | Dr Alexandre Schatz / Dr Laurence Jesel-Morel |
| 68 | GHCA - Colmar | Dr Sophie Pynn / Dr Sandrine Bellmont |
| 68 | Centre Hospitalier - Mulhouse - GHRMSA | Dr Jacques Levy / Dr Ronan Le Bouar  Dr Serban Schiau / Dr Lucien Diene |
| 69 | Hospices Civils de Lyon - GH Est-Hôpital Louis Pradel - CHU | Dr Francis Bessiere/ Dr Arnaud Dulac  Dr Philippe Chevalier / Dr Kevin Gardey |
| 69 | Clinique de l'Infirmerie Protestante de Lyon | Dr Cyril Durand / Dr Alexis Durand Dubief  Dr Hugo Brahic / Dr Hervé Poty |
| 69 | Centre Hospitalier Saint Joseph Saint Luc - Lyon | Dr Benjamin Gal / Dr Julien Pineau  Dr Samuel Chauveau |
| 69 | Clinique du Tonkin - Lyon - Villeurbane | Dr Olivier Garrier |
| 69 | Polyclinique Lyon-Nord - Rillieux | Dr Michael Attali |
| 69 | Hôpital de la Croix-Rousse - Lyon | Dr Samir Fareh / Dr Mathieu Montoy  Dr Pierre Lantelme / Dr Paul Charles |
| 71 | Centre Hospitalier Chalon sur Saône | Dr Cédric Nguyen |
| 72 | Centre Hospitalier du Mans | Dr Mathieu Amelot |
| 72 | Pôle Santé Sud - Le Mans | Dr Philippe Poret / Dr Jean Christophe Amirault |
| 73 | Centre Hospitalier de Chambéry | Dr Raoul Bacquelin |
| 74 | Centre Hospitalier Annecy Genevois | Dr Pierre Frey / Dr Didier Irles  Dr Antoine Dompnier / Dr Chrystelle Akret |
| 75 | Institut Mutualiste Montsouris | Dr Edouard Siméon / Dr Olivier Villejoubert  Dr Nicolas Mignot / Dr Pierre Jorrot |
| 75 | Hôpital St. Joseph | Dr Yamina Mouhoub / Dr Lionel Ovart  Dr Géraldine Vedrenne |
| 75 | Clinique Alleray Labrouste | Dr Jacky Ollitrault / Dr Denis Amet |
| 75 | HEGP | Dr David Perrot / Dr Emilie Varlet  Dr Pierre Baudinaud / Dr Thomas Lavergne  Dr Xavier Jouven / Dr Séverine Philibert  Dr Pauline Pinon / Dr Tej Chalbia  Dr Victor Waldmann / Dr Eloi Marijon |
| 75 | La Pitié Salpétrière | Dr Nicolas Badenco/ Dr Estelle Gandjbakhch  Dr Guillaume Duthoit / Dr Mikael Laredo  Dr Xavier Waintraub |
| 75 | Hôpital Bichat | Dr Anne Messali / Dr Antoine Leenhardt  Dr Vincent Algalarrondo / Dr Fabrice Extramiana |
| 75 | Hôpital Necker | Dr Victor Waldmann / Dr Damien Bonnet |
| 76 | CHU de Rouen | Dr Benedicte Godin / Dr Frederic Anselme  Dr Arnaud Savouré / Dr Corentin Chaumont |
| 76 | Groupe Hospitalier du Havre | Dr Nathanael Auquier / Dr Popescu Elena |
| 76 | Clinique Saint-Hilaire - Rouen | Dr Pierre Le Franc / Dr Fanny Bouchinet |
| 77 | Clinique Les Fontaines - Melun | Dr Cyrus Moini / Dr Audrey Lefoulon |
| 77 | Grand Hôpital de l'Est Francilien - Marne-La-Vallée | Dr Mohamed Belhameche / Dr Sana Sioua |
| 78 | Hôpital privé de Parly 2 | Dr Abdeslam Bouzeman / Dr Cathy Bertrand  Dr Franck Halimi |
| 78 | CHI de Poissy - Saint Germain en Laye | Dr Thomas Chastre / Dr Khadidja Belkir  Dr Raphael Gdalia / Dr Denis Amet |
| 80 | CHU Amiens | Dr Jean Sylvain Hermida / Dr Alexis Hermida  Dr Akli Otmani / Dr Maciej Kubala |
| 80 | SAS Cardiologie et Urgences - Amiens | Dr Sarah Traulle / Dr Denis Raguin |
| 81 | Centre Hospitalier d'Albi | Dr Marie Blaye-Felice / Dr Philippe Rumeau |
| 81 | Centre Hospitalier Intercommunal Castres-Mazamet | Dr Pascal Chavernac / Dr Marion Pouche |
| 82 | Centre Hospitalier de Montauban | Dr Nouredine El Hajjaji |
| 83 | Polyclinique Les Fleurs - Toulon | Dr Emilie Bastard |
| 83 | Centre Hospitalier Toulon | Dr Isabelle Lecardonnel  Dr Essia Lakhal-Ben Larbi |
| 83 | Hôpital d'instruction des armées Sainte-Anne - Toulon | Dr Gilles Cellarier / Dr Raphaël Demoulin |
| 84 | Centre Hospitalier d'Avignon | Dr Olivier Barthez / Dr Jean Paul Faugier  Dr Saida Cheggour |
| 84 | Clinique Rhône Durance - Avignon | Dr François Xavier Hager / Dr Frédéric Ortuno |
| 85 | Centre Hospitalier Départemental Vendée - La Roche-sur-Yon | Dr Olivier Billon |
| 86 | CHU de Poitiers | Dr Rodrigue Garcia / Dr Bruno Degand  Dr François Le Gal |
| 87 | CHU de Limoges | Dr Benoit Guy Moyat |
| 89 | Centre Hospitalier d’Auxerre | Dr François Jourda / Dr Stéphane Mourot |
| 90 | Hôpital Nord Franche-Comté | Dr Renaud Fouché |
| 91 | Institut Jacques Cartier - Massy | Dr Jerome Horvilleur / Dr Laurent Fiorina  Dr Jerome Lacotte / Dr Fiorella Salerno  Dr Salem Younsi / Dr Mina Ait Said  Dr Vladimir Manenti |
| 91 | Centre Hospitalier Sud Francilien - Corbeil-Essones | Dr Mohanad Mahfoud / Dr Monteau Jacques |
| 91 | Hôpital privé Claude Galien - Quincy-sous-Sénart | Dr Vladimir Manenti / Dr Dominique Bleinc |
| 92 | Clinique Ambroise Paré - Neuilly-sur-Seine | Dr Christine Alonso/ Dr Arnaud Lazarus  Dr Ghassan Moubarak / Dr Olivier Thomas  Dr Ardalan Sharifzadehgan / Dr Alexandre Zhao |
| 92 | Hôpital Antoine-Béclère - Clamart | Dr Christophe Juin |
| 92 | Hôpital d'Instruction des Armées - Percy | Dr Vincent Kanczuga / Dr Henri Broustet |
| 92 | Hôpital Marie Lannelongue - Le Plessis Robinson | Dr Nicolas Combes / Dr Alice Maltret |
| 93 | Centre Cardiologique du Nord - Saint Denis | Dr Abdelhamid Benounane / Dr Xavier Copie  Dr Olivier Piot |
| 93 | Groupe Hospitalier Intercommunal Le Raincy Montfermeil | Dr Walid Amara / Dr Vanessa Abdou  Dr Fabien Monsel |
| 94 | Hôpital Henri Mondor - Créteil | Dr Nicolas Lellouche / Dr Nathalie Elbaz  Dr Segolene Rouffiac-Noel |
| 95 | Centre Hospitalier d'Argenteuil - Argenteuil | Dr Guillaume Galidie |
| 95 | Centre Hospitalier René Dubos - Pontoise | Dr Laurentiu Dorian Nitu |
| 98 | Centre Hospitalier Princesse Grace - Monaco | Dr Gabriel Latcu / Dr Bogdan Enache |
| 98 | Centre Cardio-Thoracique de Monaco- Monaco | Dr Nicolas Hugues |
| 971 | Centre Hospitalier de Basse-Terre - Basse-Terre | Dr Isabelle Lagrenade |
| 972 | CHU Fort-de-France - Fort-de-France | Dr Fabrice Demoniere / Dr Inamo Jocelyn  Dr Andréas Müssigbrodt |
| 974 | CHU de la Réunion - Saint Pierre | Dr Olivier Geoffroy / Dr Gael Clerici  Dr François Wiart |
| 987 | Centre hospitalier de la Polynésie française - Papeete | Dr Bruno Ulmer |
| 988 | Centre Hospitalier Territorial- Nouméa | Dr Guillaume Kabalu / Dr Olivier Axler |
